# Mechanisms and cell lineages in lymphatic vascular development

**DOI:** 10.1007/s10456-021-09784-8

**Published:** 2021-04-06

**Authors:** Daniyal J. Jafree, David A. Long, Peter J. Scambler, Christiana Ruhrberg

**Affiliations:** 1 Developmental Biology and Cancer Programme, UCL Great Ormond Street Institute of Child Health, University College London, 30 Guilford Street, London, WC1N 1EH UK; 2 Faculty of Medical Sciences, University College London, London, UK; 3 UCL Institute of Ophthalmology, University College London, 11–43 Bath Street, London, EC1V 9EL UK

**Keywords:** Embryonic development, Endothelial cell, Lymphangiogenesis, Lymphvasculogenesis, Lymphatic vasculature

## Abstract

Lymphatic vessels have critical roles in both health and disease and their study is a rapidly evolving area of vascular biology. The consensus on how the first lymphatic vessels arise in the developing embryo has recently shifted. Originally, they were thought to solely derive by sprouting from veins. Since then, several studies have uncovered novel cellular mechanisms and a diversity of contributing cell lineages in the formation of organ lymphatic vasculature. Here, we review the key mechanisms and cell lineages contributing to lymphatic development, discuss the advantages and limitations of experimental techniques used for their study and highlight remaining knowledge gaps that require urgent attention. Emerging technologies should accelerate our understanding of how lymphatic vessels develop normally and how they contribute to disease.

## Introduction

The lymphatic vasculature constitutes a blind-ended vessel network that removes fluid, cells and molecules from the interstitium and returns a proteinaceous fluid termed lymph through lymph nodes into the blood vascular circulation [1, 2]. Lymphatic vessels are comprised of oak leaf-shaped lymphatic endothelial cells (LEC) that are bound by junctions with each other to surround a lumen. The lymphatic vasculature is hierarchical, beginning with blind-ended capillaries adapted for fluid, cell and molecule uptake and transitioning to larger collecting vessels with valves and mural cell coverage for unidirectional lymph transport [3]. An example is the lymphatic vasculature lining the meninges and intervertebral spaces, which clears macromolecules as well as interstitial and cerebrospinal fluid from the central nervous system [4–9]. The importance of lymphatic drainage for central nervous system function is underscored by recent findings that its disruption likely contributes to the sequalae of traumatic brain injury [10], cognitive decline and degenerative neuropathology [11, 12]. Lymphatics are also integral for immunity via immune cell, cytokine and antigen trafficking and by releasing molecules that regulate the inflammatory milieu [13–15]. Accordingly, modulating lymphatic function has therapeutic implications for a broad repertoire of pathologies including autoimmunity [16, 17], cardiovascular disease [18–22] and cancer [23, 24].

In addition to their general role in fluid uptake and immunity, lymphatics fulfil a variety of organ-specific physiological functions. For example, lymphatic vessels in the gastrointestinal system maintain gut immune homeostasis [25] whilst also transporting lipids and fat-soluble vitamins derived from the diet [26], with gut lymphatic function perturbed in obesity [27, 28]. Other examples of organ-specific lymphatic functions include the regulation of hair follicle regeneration by dermal lymphatics [29, 30] and the maintenance of total lung compliance by pulmonary lymphatics, the latter required for lung inflation at birth in preparation for breathing [31]. These key features of lymphatic vasculature highlight the importance of studying lymphatic vessel formation and the emergence of lymphatic heterogeneity during embryonic development.

Here, we review a large body of experimental evidence, which suggests that lymphatics arise by diverse cellular mechanisms from multiple cell lineages during the extensive period of organ development in the embryo. We emphasise the strengths and limitations of the experimental techniques used to arrive at this knowledge and suggest how future studies might incorporate emerging technologies to further investigate the origin of organ-specific lymphatic functions in health and disease.

## Cellular mechanisms of lymphatic development

### Techniques and models to visualise lymphatics during embryonic development

Seminal experiments to understand lymphatic development were conducted at the beginning of the twentieth century. At this time, serial histological sections of ink-injected pig, rabbit or cat embryos were observed with light microscopy to study the origins, distribution and morphology of fluid-filled lymphatic vessels [32–36]. More recently, the mouse has served as the major mammalian model organism, due to its amenability for genetic engineering and the availability of many useful molecular markers for endothelial cells, including LECs. For example, the *Prox1*^+*/lacZ*^ knock-in mouse expresses β-galactosidase (β-gal) in cells that endogenously express the transcription factor prospero homeobox protein 1 (PROX1), a key marker of LECs [37–39], and has been used to identify lymphatic vessels in tissue sections of developing embryos. Most commonly, lymphatic vessels and individual LECs are identified in tissue sections or embryo and organ wholemounts by immunostaining for antibodies raised against PROX1 as well as other LEC markers, such as vascular endothelial growth factor receptor 3 (VEGFR3; also known as FLT4), the glycoprotein podoplanin (PDPN) [40, 41] or lymphatic vessel endothelial hyaluronic acid receptor 1 (LYVE1) [42]. Though none of these molecular markers are exclusive to lymphatic vessels, in combination with one another they can be used to accurately identify LECs. More recently, wholemount immunofluorescence staining with or without tissue clearing has been combined with confocal microscopy, optical projection tomography or light-sheet fluorescence microscopy to produce three-dimensional (3D) images of lymphatic networks in intact mouse organs or entire embryos [43–45]. These modalities allow the detection of all LECs within a tissue and 3D analyses of lymphatic formation at cellular resolution, thus providing experimental advantages to ink injection, which allows the visualisation of fluid-filled vessel lymphatic networks but cannot identify single-cell precursors or newly formed lymphatic vessel segments that are not yet fluid-filled.

Many insights into the mechanisms of lymphatic development have also arisen from live imaging of developing zebrafish larvae, which are transparent and thus exquisitely suited for live imaging of dynamic cellular behaviours, including the processes that occur during lymphatic development. More specifically, using zebrafish with transgenes that express fluorescent proteins from lymphatic promoters, such as *lyve1:EGFP* or *lyve1-DsRed2* [46], allows LECs to be directly visualised at high resolution in vivo with time-lapse confocal microscopy. Such studies have shown that zebrafish possess a lymphatic system that shares key structural and functional features with its mammalian counterpart [47, 48]. Examples of functional similarities include the ability of zebrafish lymphatic vessels to clear injected dyes from extracellular spaces [9, 47–50] and the presence of collecting vessels with valves for unidirectional lymph flow [51].

By utilising the above visualisation techniques to study genetically modified mice and zebrafish, many insights into evolutionary conserved molecular and cellular mechanisms of lymphatic development have been gathered, which we discuss in this review.

### Mechanistic hallmarks of lymphatic vessel assembly in mammals

**Fig. 1:**
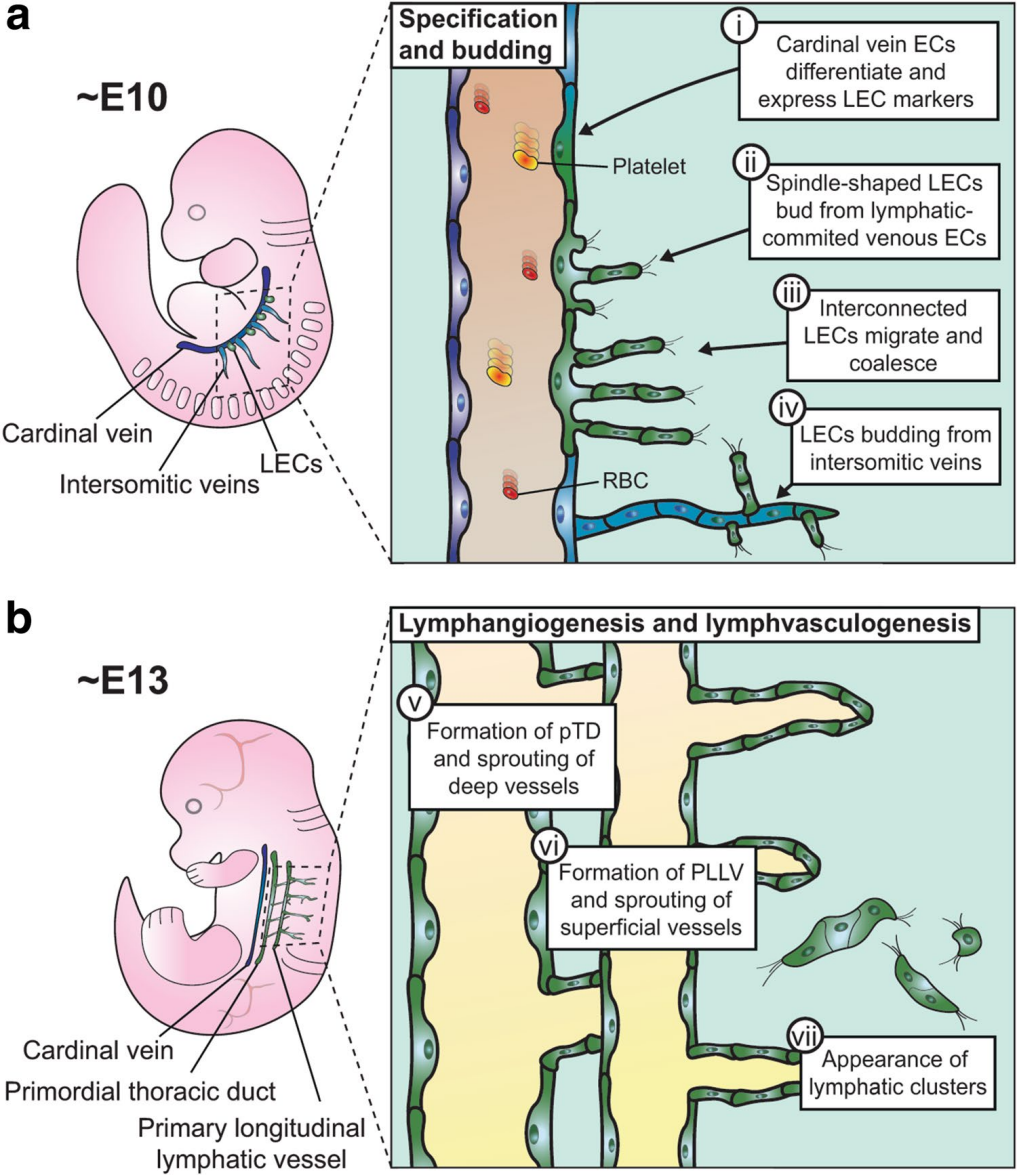
Cellular mechanisms of mouse lymphatic development. **a** *Specification and budding*: (i) From E10.0, a subset of endothelial cells in the cardinal vein differentiates towards a lymphatic identity; (ii) LECs emerge from the cardinal vein as spindle-shaped cells; (iii) interconnected LECs migrate from the cardinal vein to form the primordial thoracic duct; (iv) LECs also emigrate from the intersomitic veins. **b** *Lymphangiogenesis and lymphvasculogenesis*: (v) LECs emerging along the length of the cardinal vein have condensed into the primordial thoracic duct, from which lymphatic vessels sprout towards the viscera, and they have also condensed more laterally into the primary longitudinal lymphatic vessels, from which lymphatic vessels sprout to form a superficial lymphatic plexus; (vii) clusters of LECs arise in different tissues and contribute to lymphatic vessel formation

Based on work using the experimental approaches described above, it is now widely accepted that lymphatic vessels in mammals arise through several distinct but complementary cellular mechanisms (Fig. 1). These events are instructed by several key molecules, whose specific roles are described below (see also Table 1).

**Table 1:** Key molecules in mouse lymphatic development

Molecule	Venous specification	Lymphangiogenesis	Lymphvasculogenesis
ADAMTS3	No role established	Required for lymphangiogenesis by facilitating proteolytic cleavage of VEGFC via CCBE1 [74–76]	No role established
CCBE1	No role established	Required for budding and sprouting of LECs from veins [44] by facilitating proteolytic cleavage of VEGFC [74, 75, 77]	Required for the exit of lymphatic cluster-forming LECs from the cervicothoracic dermal capillary plexus [78]
COUP-TFII	Directly binds *Prox1* and promotes its activation for LEC differentiation [55, 58]	Regulates *Nrp2* transcription, required for LECs sprouting and proliferation, and required to maintain LEC fate [73]	No role established
FLCN	Supresses nuclear translocation of TFE3, which binds to *Prox1* and stimulates LEC differentiation [59]	No role established	No role established
GATA2	No role established	Regulates migratory responses in LECs and directly regulates VEGFR3 expression [70]	No role established
LYVE1	No role established	Not absolutely required for lymphangiogenesis [60]	No role established
NRP2	No role established	Facilitates lymphatic sprouting as a co-receptor for VEGFR3 in VEGFC signalling [71, 72]	No role established
PDPN	No role established	Involved in the platelet-mediated separation of lymphatics from the blood vasculature during lymphatic sprouting [160]	No role established
PROX1	Represses blood endothelial identity and induces LEC fate [37, 38]	Acts in a feedback loop with VEGFR3 and required to maintain LEC specification [39, 69]	Expressed in cluster cells [8, 43, 79, 81, 82] and deletion in the second heart field lineage causes ventral cardiac lymphatic agenesis [99], therefore is likely required for lymphatic cluster formation
SOX18	Promotes *Prox1* expression in venous ECs and induces LEC fate specification [57]	No role established	No role established
VEGFC	No clear role as VEGFC mutants have lymphatic progenitors in the wall of the cardinal vein [44, 66]	Essential for the sprouting of LEC progenitors from venous endothelium LECs [44, 66]	Promotes lymphatic cluster expansion and number [43, 78]
VEGFR3	Acts in a feedback loop with PROX1 to maintain LEC identity whilst in the wall of the vein [69]	Acts cell-autonomously and non-cell-autonomously for LEC proliferation, migration and survival [67–69]	No role established

*ADAMTS3* A disintegrin and metalloprotease with thrombospondin motifs-3, *CCBE1* collagen and calcium-binding EGF domain-containing protein 1, *COUP-TFII* COUP transcription factor 2, *LYVE1* lymphatic vessel endothelial hyaluronan receptor 1, *EC* endothelial cell, *FLCN* folliculin, *GATA2* GATA-binding factor 2, *LEC* lymphatic endothelial cell, *NRP2* neuropilin 2, *PDPN* podoplanin, *PROX1* prospero homeobox protein 1, *SOX18* sex determining region Y box 18, *TFE3* transcription factor E3, *VEGFC* vascular endothelial growth factor C, *VEGFR3* vascular endothelial growth factor receptor

#### Venous specification

Early experiments in pig and rabbit embryos led to the ‘venous’ model of lymphatic development, based on the observation that ink injection of superficial lymphatic vessels traced connections to blind ducts budding from venous endothelium [32–35]. Subsequently, multiple studies have used histological techniques in mice to confirm that lymphatic development begins by venous specification and pinpointed this to occur between embryonic day (E)9.5 and 10.0, shortly after the specification of the major arteries and veins [52] and around the timing of cardiac septation [53] (Fig. 1a). These studies employed immunostaining of tissue sections or wholemount preparations from *Prox1*^+*/lacZ*^ or wildtype embryonic mice to demonstrate that a subpopulation of cells in the anterior wall of the cardinal vein and the adjacent intersomitic veins begin to express PROX1 [37, 38, 44, 54]. In this subpopulation of venous cells, PROX1 is co-expressed with two other transcription factors, sex determining region Y box (SOX)18 and chicken ovalbumin upstream promoter transcription factor II (COUP-TFII, also known as NR2F2). These transcription factors interact with one another to drive venous endothelial cells to differentiate into lymphatic progenitors [55–58]. The commitment of venous cells to a LEC fate is negatively regulated by folliculin (FLCN), which prevents the accumulation and nuclear translocation of the transcription factor E3 (TFE3) that promotes *Prox1* expression [59]. Accordingly, loss of FLCN causes excessive commitment of venous endothelial cells to LECs and also promotes LEC proliferation [59]. Whilst within the wall of the cardinal vein, lymphatic progenitors also begin to express LYVE1, which serves as a useful molecular marker for lymphatic vessels, but is not known to be required for the venous specification of endothelium [60] (Table 1).

#### Lymphangiogenesis

At the next stage of lymphatic development, lymphatic progenitors exit from the cardinal vein and coalescence to form the first lymphatic vessels (Fig. 1b). This process was first observed in immunolabelled tissue sections of E10.5 *Prox1*^+*/lacZ*^ and wildtype mouse embryos [37, 38]. By E11.5, these progenitors have migrated dorsally and anteriorly as interconnected cells and condense into structures adjacent to the cardinal vein that appear as sacs when viewed in tissue sections [45, 54]. This process was subsequently re-evaluated by high-resolution 3D imaging of immunolabelled and optically cleared mouse embryos with light-sheet fluorescence microscopy [44]. These experiments showed that lymphatic progenitors sprout from along the length of the cardinal vein as groups of spindle-shaped cells, and that they condense into the continuous primordial thoracic duct, the main axial lymphatic vessel, and the paired peripheral longitudinal lymphatic vessels, rather than discrete lymph sacs [44]. Sprouting of LECs dorsally from the peripheral longitudinal lymphatic vessels was further shown to give rise to a superficial lymphatic plexus [44]. The process of lymphatic sprouting from pre-existing lymphatic vessels has been termed lymphangiogenesis [61], in analogy to the angiogenic process by which blood vessels sprout [62]. In both angiogenesis and lymphangiogenesis, filopodia-studded endothelial cells lead new vessel sprouts that emerge from a pre-existing vascular network in which endothelial cells proliferate.

As lymphangiogenesis was initially thought to be the predominant mechanism by which lymphatic vessels arise [63], the molecules driving this process have been extensively studied using genetic knockout experiments (Table 1). Whilst blood vessel angiogenesis is a VEGFR2-driven response of endothelial tip cells [64] to VEGFA gradients [65], lymphangiogenesis involves a VEGFR3 response to VEGFC signals [66–68]. Mesenchyme adjacent to the cardinal vein secretes VEGFC [66], which binds to VEGFR3 on the cardinal vein to promote the sprouting, proliferation, migration and survival of LECs [68, 69]. Several molecules promote VEGFC signalling in LECs. To increase VEGFC responsiveness, the transcription factor GATA-binding factor 2 (GATA2) promotes VEGFR3 expression in LECs as they exit the cardinal vein [70]. Further, neuropilin (NRP)2 acts as a co-receptor for VEGFR3 to promote VEGFC-dependent lymphatic sprouting [71, 72], whereby NRP2 expression is up-regulated by COUP-TFII [73]. The secreted protein collagen and calcium binding EGF domains 1 (CCBE1) activates the A disintegrin and metalloproteinase with thrombospondin motifs 3 (ADAMTS3), which in turn promotes the proteolytic cleavage of the otherwise poorly active 29/31 kDa form of VEGFC to its active 21/23 kDa form [74–77].

#### Lymphvasculogenesis

**Fig. 2:**
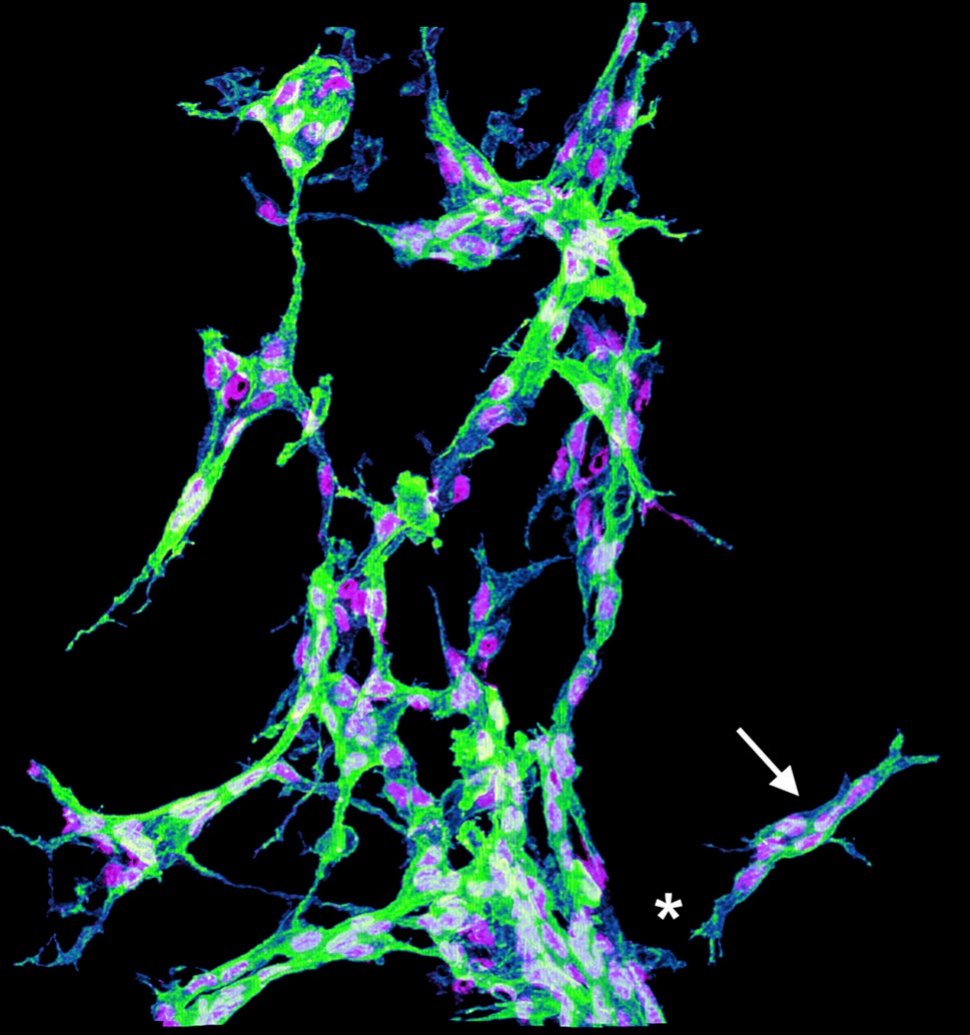
3D imaging of lymphangiogenesis and lymphvasculogenesis in kidney lymphatic development. The image shows a 3D reconstruction of a confocal *z-*scan though the hilar region of a E15.5 mouse embryonic kidney, stained for LYVE1 (green; labelling LEC membranes) and PROX1 (magenta; labelling LEC nuclei). Segmentation of lymphatic structures using 3D imaging software reveals a lymphatic cluster (arrow) adjacent to the sprouting lymphatic vessel plexus (asterisk). The image was produced using an LSM880 confocal microscope (Zeiss) with an Airyscan detector, segmented using IMARIS (Bitplane) and edited using FIJI (NIH)

Combining wholemount immunostaining with confocal imaging of developing mammalian organs brought to light a complementary cellular mechanism of lymphatic development. In particular, imaging of developing mouse skin [78–80], mesentery [81] heart [82], lung [83], intestine [84] meninges [8, 85] and kidney [43] revealed islands of individual LECs that appeared to coalesce into lymphatic vessels (Fig. 2). Lymphatic clusters in the mesentery, meninges and dermis contain proliferating cells [8, 78, 81] and cluster cells extend protrusions towards other clusters or nearby lymphatic vessels [8, 43, 78, 79, 81] (Fig. 2). These protrusions might function similarly to filopodia on blood vessel tip cells to initiate fusion [62]. Quantitative analysis of kidney, mesentery and dermis showed that the abundance of clusters declines as the lymphatic plexus expands through gestation [8, 43, 78]. Altogether, these findings suggest that cluster LECs proliferate and fuse, both with each other and to pre-existing lymphatic vessels, to expand organ lymphatic networks. This process has been termed lymphvasculogenesis [3] due to its similarity to vasculogenesis, a process by which blood vascular endothelial cells differentiate from single cell precursors and then coalesce. Notably, lymphatic clusters without discernible physical connections to sprouting lymphatic vessels were also observed in the human embryonic kidney [43], suggesting that lymphvasculogenesis is also an important mechanism for human lymphatic development, albeit other human organs have not yet been examined for such structures. Given its recent discovery, the molecular mechanisms underpinning lymphvasculogenesis remain poorly understood, but recent studies have begun to identify molecules that affect lymphatic cluster formation (Table 1). For example, it was shown that CCBE1 loss abrogates dermal lymphatic cluster formation in mice, whereas this process was increased by endothelial VEGFC overexpression [78]. VEGFC is therefore likely a universal regulator of LEC proliferation. An area that warrants further study pertains to evidence suggesting that sex [19] and genetic background [43, 57, 86] could affect lymphangiogenesis and lymphvasculogenesis in mice.

### Mechanistic hallmarks of lymphatic vessel assembly in zebrafish

**Fig. 3:**
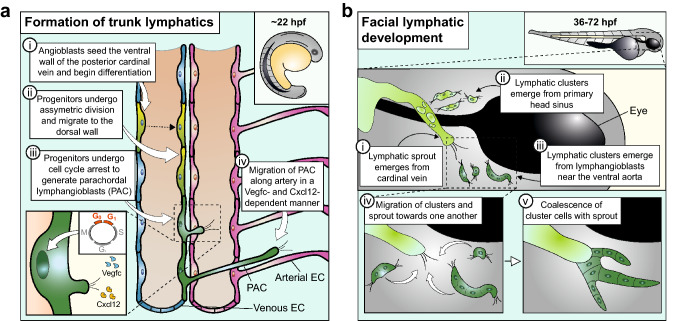
Insights from zebrafish into the cellular dynamics of lymphatic development. **a** *Formation of trunk lymphatic vessels:* (i) A subset of angioblasts seeds the floor of the cardinal vein to give rise to endothelial cells, which begin to adopt a LEC identity from 22 hpf onwards; (ii) these progenitors undergo asymmetric division and give rise to lymphatic progenitors that migrate to the dorsal wall of the vein (the dotted line indicates cell division); (iii) these lymphatic progenitors give rise to parachordal lymphangioblasts (PAC); (iv) the PACs sprout along adjacent arterial endothelial cells (EC). The inset shows that lymphatic progenitors in the cardinal vein undergo cell cycle arrest and give rise to PACs that sprout, driven by gradients of Vegfc and the chemokines Cxcl12a and Cxcl12b. **b** *Formation of facial lymphatic vessels:* (i) Facial lymphatic sprouts emerge from the cardinal vein by 36 hpf; (ii) lymphatic clusters emerge from the primary head sinus by 48 hpf and fuse with the cardinal vein-derived lymphatic sprout; (iii) lymphatic clusters arise adjacent to the ventral aorta by 72 hpf and (iv) migrate towards and (v) fuse with the growing venous-derived lymphatic sprout to form the facial lymphatic network

Live imaging during early lymphatic development in zebrafish has provided novel insights into the cellular dynamics of venous specification and lymphangiogenesis (Fig. 3a). For example, time-lapse imaging of transgenic zebrafish expressing fluorescent proteins controlled by the promoter of *prox1a*, an orthologue of mammalian *Prox1*, localised the first lymphatic progenitors to the ventral wall of the posterior cardinal vein at 22–24 h post-fertilisation (hpf) [87, 88]. These progenitors undergo asymmetric division and translocate to the dorsal wall of the vein [87, 88]. Live imaging of a *fucci* reporter, which identifies cells in specific stages of the cell cycle, showed that lymphatic progenitors undergo cell cycle arrest prior to emigrating from the posterior cardinal vein [89]. Upon their exit from the vein, LECs migrate along intersomitic arterial vessels, in a manner dependent on Vegfc [47, 48, 87, 90] and Cxcl12a and Cxcl12b gradients [91], and form the lymphatics of the zebrafish trunk via lymphangiogenesis [47, 48, 50]. These cellular dynamics largely agree with the mechanisms inferred from fixed mouse tissues.

At later stages, time-lapse imaging of the developing facial lymphatic network has demonstrated that lymphvasculogenesis also occurs in fish to complement lymphangiogenesis (Fig. 3b). Live imaging of *lyve1:EGFP* transgenic zebrafish embryos showed that facial lymphatic vessels sprout from the cardinal vein from 36 hpf and fuse with lymphatic clusters that arise from the primary head sinus at 48 hpf. Additionally, a different population of lymphatic clusters arises at 72 hpf adjacent to the ventral aorta, and these clusters migrate towards and coalesce with venous-derived lymphatic vessels [46, 92].

Despite overall conservation of key mechanisms in lymphatic development between mouse and zebrafish, the genetic knockdown of some genes implicated in lymphatic development in mouse cause only mild lymphatic phenotypes in zebrafish [87, 93, 94]. Such differences might be explained by the persistence of maternal transcripts that reduce the penetrance of genetic mutations in the zebrafish [87] or the presence of duplicated genes in the zebrafish genome which compensate for one another [95, 96]. Alternatively, different phenotypes may be due to evolutionary divergence in the requirement of certain molecules for lymphatic development [97].

## Cell lineages contributing to lymphatic endothelium

**Fig. 4:**
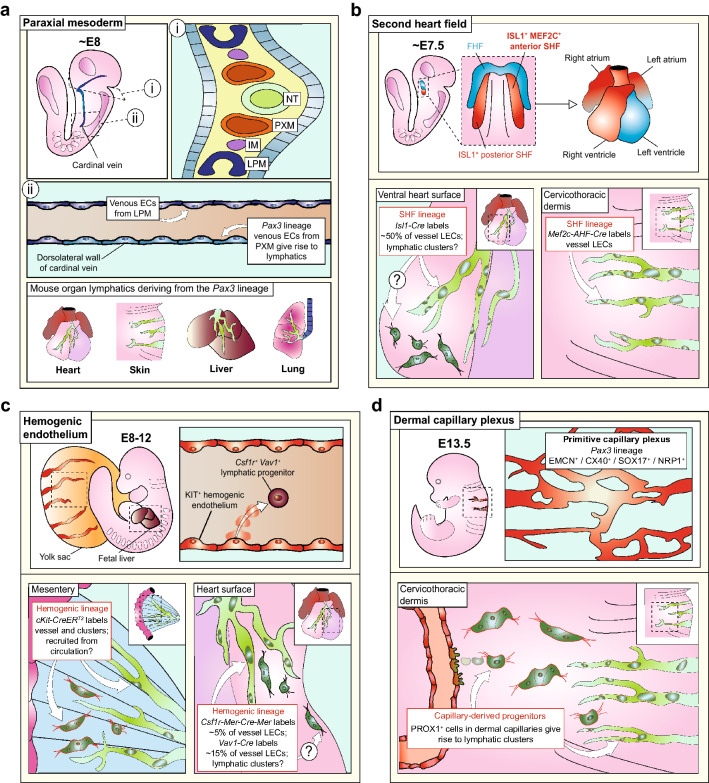
Cellular lineages contributing to lymphatic endothelium in mouse. **a** *Paraxial mesoderm:* (i) The paraxial mesoderm (PXM) is a transient cell population located between the intermediate mesoderm (IM) and the neural tube (NT) and gives rise to muscle lineages and limb endothelium; (ii) based on lineage tracing from the *Pax3* promoter*,* the PXM is proposed to contribute venous endothelial cells (ECs) to the dorsolateral wall of the cardinal vein, whereas the rest of the vein is derived from lateral plate mesoderm (LPM). Thereby, the *Pax3* lineage gives rise to most LECs in the heart, liver and lung as well as thoracic, lumbar and sacral skin. **b** *Second heart field:* The cells of the second heart field (SHF), located adjacent to the first heart field (FHF), contribute to the poles of the developing heart. Based on lineage tracing with the *Isl1* and *Mef2c* promoters, LECs from the SHF are proposed to contribute LECs to the ventral surface of the heart and the cervicothoracic region of the dermis. It is not yet known whether the SHF lineage contributes cells to lymphvasculogenesis in the heart. **c** *Hemogenic endothelium:* Hemogenic endothelia in the yolk sac and several intraembryonic sites give rise to circulating KIT+ progenitors with the potential to differentiate into erythrocytes and myeloid cells. Based on lineage tracing from the KIT promoter using an inducible *Kit-CreER*^*T2*^ allele, hemogenic EC-derived progenitors are proposed to give rise LECs in lymphatic vessels and clusters of the mesentery. Based on lineage tracing from the haematopoietic *Csf1r* and *Vav1* promoters, hemogenic EC-derived progenitors are proposed to give rise to LECs within cardiac lymphatic vessels, but the contribution of these lineages to lymphatic clusters in the heart has not yet been examined. **d** *Dermal capillary plexus:* Based on lineage tracing from the *Pax3* promoter*,* the PXM is proposed to contribute to dermal blood vascular capillaries which then give rise to dermal LECs via a VEGFC/CCBE1-dependent budding mechanism

Whereas lymphangiogenesis of venous-derived LECs was initially considered the predominant mode of lymphatic development [63], the discovery of lymphvasculogenesis has prompted re-examination of the cellular origins of LECs in recent years. Experiments facilitated by genetic lineage tracing suggest that paraxial mesoderm is the precursor of venous-derived LECs in the mouse [80] and that non-venous progenitors provide additional cellular LEC sources for organ lymphatic formation (Fig. 4). Before we discuss these findings in more detail, we provide an overview of the main experimental strategy for genetic lineage tracing of mammalian lymphatics, which utilises the Cre-loxP recombination system in mouse.

### Lineage tracing lymphatic origins via Cre-loxP recombination in mouse

The Cre enzyme from the P1 bacteriophage is a site-specific recombinase that excises DNA segments flanked by loxP sites [98]. For genetic lineage tracing, Cre is expressed under the control of a promoter that drives cell-type specific gene expression, either from a transgene or when knocked directly into the endogenous locus. This strategy is used to recombine reporter genes, in which a loxP-flanked stop codon precedes the coding sequence of a non-mammalian protein, such as β-galactosidase, or the fluorescent proteins GFP and tdTomato. Accordingly, the reporter protein is only present in Cre-expressing cells and their descendants to facilitate lineage tracing.

The *Tie2-Cre* transgenic mouse has been used for genetic lineage tracing to demonstrate the venous origin of LECs during early lymphangiogenesis [63] as well as during cardiac [22, 99] and dermal [78, 79] lymphatic development. This strategy was based on the observation that transcripts for tyrosine-protein kinase receptor (TIE)2 are enriched in venous relative to arterial endothelium [100]. Moreover, in situ hybridisation for *Tie2* and immunostaining of *Tie2-GFP* mice have not detected *Tie2* expression in LECs budding from the cardinal vein or lymphatic vessels of E11.5 to E15.5 embryos [63, 78]. When *Tie2-Cre* mice are bred with a *R26R* reporter line, the encoded β-galactosidase is active in the anterior cardinal vein, in LECs budding from the cardinal vein at E11.5 and in PROX1^+^ cells of adjacent lymph sacs at E13.5 and E14.5 [63]. These studies therefore corroborate that LECs can be derived from a *Tie2* lineage of venous progenitors. In the future, recently identified venous-specific promoter or enhancer sequences [101] may provide alternative, more specific lineage tracing tools for venous endothelial derivatives. Notably, TIE2 has been detected by immunostaining in adult mouse ear LECs [102]. Therefore, it will be important to determine whether LEC labelling with *Tie2-Cre* can be caused by TIE2 expression in LECs in some organs.

Temporal control was subsequently introduced into the Cre-loxP system by fusing a modified estrogen receptor ligand binding domain to Cre (CreER), so that Cre is retained in the cytoplasm until bound by an ER agonist, typically hydroxytamoxifen (4-OHT); in its agonist-bound form, Cre is then translocated to the nucleus as a prerequisite for Cre-induced genomic DNA recombination [98]. The most popular version of CreER, CreER^T2^, carries point mutations that reduce binding of endogenous estrogen. The promoters of the endothelial genes encoding platelet-derived growth factor (PDGF)B [22, 81], apelin receptor (APJ) [82], cadherin 5 (CDH5) or SOX18 [78] have all been used for lineage tracing of LECs, whereby 4-OHT or its precursor, tamoxifen, are administered to mice pregnant with embryos carrying both CreER^T2^ and a Cre-dependent reporter.

CreER^T2^-based genetic lineage tracing can be performed in specific gestational windows to discriminate between different cellular origins of lymphatics based on their temporal emergence. For example, a tamoxifen pulse administered to activate *Vegfr3-CreER*^*T2*^ in E14.5 mouse embryos labelled LECs in both the dorsal and ventral heart at E17.5 [103]. By contrast, an earlier tamoxifen pulse at E11.5 or E12.5 predominantly labelled dorsal, but not ventral LECs, suggesting that ventral LECs arise at a later timepoint [103]. Further, administration of 4-OHT to E15.5 embryos carrying *Prox1-CreER*^*T2*^ labelled established dermal lymphatic vessels at E17.5, but lymphatic clusters in the midline were not labelled; these findings suggest that lymphangiogenesis and lymphvasculogenesis in the dermis occur from mutually exclusive cell lineages [79]. The duration of Cre recombination in these experiments, however, must be considered when interpreting the findings, because the estimated half-life of tamoxifen in mice ranges from 6 to 16 h, depending on the route of administration and dose, whilst 4-OHT clearance occurs in a shorter timeframe [79, 104–106]. Thus 4-OHT administration may be desirable to capture a lineage within a short developmental window, whereas tamoxifen might be appropriate for maximal recombination over a longer timeframe.

Despite the advantages of the Cre-loxP system to identify cell lineages, the complexity of gene expression patterns during embryonic development makes it unlikely that the expression of a single gene accurately demarcates any cell lineage with high specificity; this caveat, in turn, will generate some ambiguity when interpreting reporter labelling to deduce lymphatic origins. For example, TIE2, PDGFB and CDH5 are not specific to veins, but are also expressed in capillaries and hemogenic endothelium [107–109]. This knowledge is pertinent, given the deduction that non-venous endothelium is a putative source of lymphatics in multiple mouse organs [22, 78, 79, 81]. Moreover, LECs themselves express CDH5, and some LECs also express PDGFB [54, 110–113], raising the possibility that some lymphatic recombination after *Cdh5-CreER*^*T2*^ and *Pdgfb-CreER*^*T2*^ activation can be attributed to Cre expression in LEC themselves.

Other limitations of the Cre-loxP system include potential ectopic Cre activity when transgenes containing Cre insert into genetic loci that inadvertently modify transgene promoter activity, or when recombination unexpectedly occurs in the germline [114, 115]. Spontaneous CreER^T2^ translocation to the nucleus, in the absence of tamoxifen, may also confound the interpretation of lineage tracing studies [116]. Incomplete recombination due to suboptimal 4-OHT administration or different bioavailability in different organs [117, 118] may also affect the efficiency of genetic lineage tracing. Conversely, deleterious effects to the embryo occur from the toxicity of high levels of tamoxifen, 4-OHT or their delivery vehicles [119–121]. Toxicity can also arise when Cre recognises genomic sequences resembling loxP sites to cause DNA breaks [122], especially when Cre is expressed highly, or when random genomic insertion of transgenes disrupts essential endogenous genetic loci [123]. Unexpected phenotypes arising from these deleterious effects, which may vary according to the genetic background of the mice, may further affect the interpretation of lineage tracing studies. Although specific examples of these scenarios have not yet been reported for lymphatic development, tamoxifen-induced CreER activation impairs postnatal blood vascular growth [124]. The limitations of Cre-mediated genetic lineage tracing and potential solutions to improve studies involving this technique are listed in Table 2.

**Table 2:** Limitations of Cre-loxP lineage tracing in mouse and potential solutions

Problem	Possible reasons	Solution
Unexpected cell types captured by lineage trace	Expression of gene promoter driving Cre occurs in other, off-target cell types	Thorough characterisation of expression of gene driving Cre expression or its product
	Ectopic activity of Cre transgene due to modification of promoter activity in genetic locus	Use of other complementary Cre drivers to validate results; use of alternative transgenes or knock-in alleles
	Spontaneous translocation of CreER to nucleus in the absence of tamoxifen	Comparison with vehicle-only controls
	Spontaneous recombination of reporter alleles in the absence of Cre	Quantification of recombination in the absence of Cre; use of alternative and complementary reporter alleles
	Unexpected recombination of Cre in the germline	Inheritance of Cre from alternative sex or use of inducible CreER
Target cell population not captured by lineage trace	Inefficient recombination of CreER alleles	Increase tamoxifen or 4-OHT dose, selection of alternative CreER line or use of constitutively active Cre
	Time-window of tamoxifen or 4-OHT and CreER activity not coincident with target cell population	Optimising of timing and dosage of tamoxifen or 4-OHT
	Poor bioavailability of 4-OHT or CreER in target tissue	Using alternative method of tamoxifen or 4-OHT delivery; increase tamoxifen or 4-OHT dose
Phenotype or abortion of lineage traced embryos	Random insertion of transgene into critical genetic locus	Comparison with appropriate controls lacking Cre expression; selection of alternative Cre line
	High levels of Creor CreER induction causing DNA damage	Ensure Cre-expressing embryos are heterozygous or hemizygous and titrate tamoxifen or 4-OHT levels to limit CreER activation
	Haploinsufficiency due to heterozygous loss of function in knock-in allele	Comparison with appropriate controls lacking Cre expression; selection of alternative Cre line
	Teratogenicity of tamoxifen or 4-OHT; detrimental effect of vehicles on pregnancy (e.g., peritoneal inflammation)	Titration of tamoxifen or 4-OHT dosage by weight or age of reporter mouse; co-administration of progesterone for early pregnancy; reduce volume of vehicle used for delivery

### Defining how venous-derived lymphatics are first specified

The first intra-embryonic endothelial progenitors are thought to arise from hemangioblasts in lateral plate mesoderm [125], which then condense as blood vascular endothelial cells into the paired dorsal aortae and cardinal veins from approximately E7.5 onwards [126]. Therefore, it has been hypothesised that lateral plate mesoderm might be the source of the first LECs that emerge from the cardinal vein. Zebrafish experiments supporting this hypothesis have taken advantage of a photoconvertible Kaede protein expressed under the control of the promoter for *kinase insert domain receptor like* (*kdrl*), which encodes an endothelial VEGF receptor that is not orthologous to mammalian KDR [88]. A pulse of ultraviolet light photoconverts Kaede from green to red, and the immediate progeny of photoconverted cells, which inherit the protein, also remain red, thus providing a means of evaluating cell lineage. Photoconversion of *kdrl*-expressing cells located lateral to the dorsal aorta, a region containing presumed lateral plate mesoderm-derived angioblasts [95], also labelled lymphatic progenitors in the zebrafish trunk [88]. This finding was interpreted to suggest that lymphatics are derived from lateral plate mesoderm. However, is not clear whether this approach can distinguish cells arising from lateral plate mesoderm or from other mesodermal sources nearby. Experiments involving homotypic transplantation from quail into chick embryos refute that lateral plate mesoderm is the origin of lymphatics in avian species. Specifically, LECs within the jugular region of the developing chick wing were not found to express quail antigens after transplantation of lateral plate mesoderm from quail [127], but did so after transplantation of somites [128].

The concept that somitic precursor cells give rise to LECs has gained support from a recent mouse study, in which Cre was constitutively expressed from the promoter of paired box protein (*Pax)3* [80]. This study reported that endothelial cells in the dorsolateral wall of the cardinal vein at E9.5 were derived from a *Pax3* lineage, as were LECs emigrating from the cardinal vein at E10.5 [80]. As PAX3 protein is expressed in the paraxial mesoderm that gives rise to somitic cells, but is not present in the endothelial cells themselves [80], it was concluded that venous LEC progenitors arise from the paraxial mesoderm (Fig. 4a). *Pax3* lineage tracing further suggested that the vast majority of dermal and cardiac LECs derive from paraxial rather than lateral plate mesoderm [80], albeit the proportion of *Pax3* lineage-derived LECs was not quantified for these organs. The *Pax3* lineage also included LECs in lumbar clusters [80], raising the possibility that the paraxial mesoderm also gives rise to LECs via lymphvasculogenesis.

Several challenges still remain. Firstly, additional work is required to demonstrate that lack of PAX3 protein in endothelial cells extends to transcript levels, as a prerequisite to exclude *Pax3* promoter activity in endothelial cells themselves. Secondly, it should be considered that *Pax3*-mediated constitutive Cre expression is not exclusive for detecting paraxial mesoderm derivatives. Thus, it is important to identify all embryonic progenitor populations captured by the *Pax3* lineage and perform complementary lineage tracing approaches. Such lineage tracing studies should include inducible CreER approaches to narrow down the developmental window during which LEC precursors express *Pax3*. Aditionally, it would be desirable to corroborate that paraxial mesoderm is the only *Pax3*-expressing population that gives rise to LECs to determine if paraxial mesoderm is the only source of *Pax3*-expressing LEC progenitors. For example, neural crest cells also express PAX3, but these cells do not give rise to lymphatics, because the pan-neural crest promoter *Wnt family member (Wnt) 1* did not capture LECs, at least in the heart [22]*.* Similar experiments might be performed for other candidate *Pax3* expressing cell lineages, should they exist. Another challenge is to identify the origin of LECs that are not captured by the *Pax3* lineage, including the lymphatics of the meninges, ear skin, mesentery and intestines [80].

### Non-venous origins of lymphatic endothelial cells

Although *Pax3* lineage tracing suggested that the vast majority of LECs are derived from paraxial mesoderm, the second heart field [80, 99, 103], hemogenic endothelium [22, 81] and blood capillaries within the dermis [78] have also been proposed to contribute to the lymphatic vasculature.

#### Second heart field

During cardiac development, the elongation of the primitive heart tube and eventual septation of the heart requires a contribution from extracardiac progenitor cells in the lateral plate mesoderm. This population is termed the second heart field and is a source of smooth muscle, endothelial and myocardial cells for the arterial and venous poles of the developing heart [129]. Recently, several genetic lineage tracing studies suggested that a second heart field progenitor contributes to cardiac lymphatics (Fig. 4b).

Two lymphatic lineage tracing studies used a constitutively active *insulin gene enhancer protein (Isl1)-Cre*, which is expressed in the second heart field [130]. These studies suggested that half of all LECs on the ventral cardiac surface have a different origin [99, 103]. These conclusions were strengthened when inducible *Isl1-CreER* was tamoxifen-activated at E8.5, when the second heart field has formed but before the onset of LEC differentiation, because ventral cardiac LECs were also lineage-traced [103]. However, ISL1 is also expressed in pharyngeal and foregut endoderm at E8.5 [130], and it was therefore important that the second heart field origin of cardiac LECs was corroborated with a constitutively active Cre expressed under the control of a *myocyte enhancer factor 2c* (*Mef2c*) regulatory region that confers specific expression to the anterior region of the second heart field (*Mef2c-AHF-Cre*) [131]. These experiments showed that the *Mef2c* lineage includes a small proportion of LECs on the ventral, but not the dorsal surface of the mouse embryo heart [80, 103]. However, further investigation is required to determine why a smaller proportion of LECs is detected with *Mef2c-AHF-Cre* compared to *Isl1-Cre*.

It further remains to be established whether second heart field-derived progenitors undergo lymphvasculogenesis to form the lymphatic clusters recently described in the mouse embryonic heart [82], or whether there is an intermediate cell population, such as second heart field-derived blood endothelial cells [132], which secondarily gives rise to cardiac LECs, akin to the venous [63] or capillary [78] origin of lymphatics in other parts of the embryo. Further, it has to be considered that lymphatic vessels in the cervical skin, jugular and facial regions also contain LECs that are lineage-traced by *Mef2c-AHF-Cre* and *Isl1-Cre* [80, 99]. Therefore, it would be important to ascertain that these transgenes are not active in venous endothelium, paraxial mesoderm or other cell types with the potential to form lymphatics.

#### Hemogenic endothelium

During mammalian development, blood vessels in the yolk sac, umbilical vessels and aorta-gonad-mesonephros region give rise to so-called hemogenic endothelial cells, which generate circulating hematopoietic progenitors with the potential for myeloid, lymphoid, erythroid and blood endothelial differentiation [133]. Although lineage tracing of hemogenic endothelium is challenging due to shortcomings of individual tools used to trace its derivatives, the collective results obtained by lineage tracing with multiple different inducible and constitutively active Cre lines implicates circulating progenitors from hemogenic endothelium as a source of LECs in the heart [22] and mesentery [81] (Fig. 4c).

An inducible Cre knock-in allele utilising the promotor of the hemogenic endothelial cell surface protein KIT (*Kit-CreER*^*T2*^), when induced with tamoxifen at E10.5, labelled lymphatic clusters in the E13.5 mesentery, but only in 29% of embryos [81]. Low labelling efficiency might be explained by inefficient recombination. In agreement, we found that the *Kit-CreER*^*T2*^ allele requires high doses of tamoxifen or 4-OHT for efficient activity [133]*.* Notably, it has recently been reported that *Kit* is also expressed in some non-hemogenic blood vascular endothelial cells at E12.5 [134], which may have been captured when a high dose of tamoxifen is given at E10.5. Moreover, it is not clear when during development *Kit* is first expressed in a subset of non-hemogenic blood vascular endothelial cells. Further knowledge of the precise spatiotemporal *Kit* expression pattern in blood and lymphatic endothelia and a refined time window of *Kit-CreER*^*T2*^ induction might therefore help to demonstrate specificity of individual *Kit*-based lineage tracing for hemogenic endothelia and therefore to corroborate the origin of LEC subsets from this source.

In another study, a constitutively active Cre under the control of the promoter for the gene encoding the hematopoietic progenitor marker Vav guanine nucleotide exchange factor 1 (VAV1) was found to capture 15% of cardiac LECs [22], therefore supporting the hemogenic origin of a subset of LECs. As VAV1 is expressed in hematopoietic progenitors after budding from hemogenic endothelium [135], this approach may not have captured all hemogenic endothelium-derived LECs. In support of this idea, *Vav1*-*Cre* did not label mesenteric LECs [81]. Similarly, lineage tracing studies utilising tamoxifen-inducible lineage tracing from the promoter of the gene encoding the colony stimulating factor 1 receptor (CSF1R), active in hemogenic endothelium-derived progenitors, labelled only 5% of cardiac LECs [22]. Such limitations, in addition to the temporally overlapping hemogenic endothelial activity of the yolk sac, umbilical vessels and aorta-gonad-mesonephros [136], have obscured the precise identity and prevalence of hemogenic endothelial progenitors for LECs.

#### Blood capillaries

Blood capillaries have recently been proposed to give rise to dermal LECs in the cervical and thoracic regions (Fig. 4d). Inducing *Prox1-CreER*^*T2*^ with 4-OHT at E12.5 identified a rare population of PROX1-expressing LEC progenitors within the walls of dermal capillaries [78]. Further lineage tracing with *Cdh5-CreER*^*T2*^ identified a population of *Cdh5* lineage blood capillary endothelial cells expressing PROX1 [78]. The authors further found that VEGFC promoted LEC exit from dermal capillaries, and that CCBE was required for this process [78]. By inducing the pan-endothelial *Sox18-CreER*^*T2*^ transgene at E9.5, when the first lymphatic progenitors are specified in the cardinal vein, the authors lineage-labelled dermal LECs both in lymphatic clusters as well as lymphatic vessels of the cervical and thoracic regions [78]. A similar result was obtained with *Tie2-Cre*, despite the absence of detectable TIE2 expression in dermal LECs [78]. These findings led the authors to conclude that dermal blood capillaries contain LEC progenitors, and that these are capable of giving rise to lymphatic clusters, akin to the LECs that arise in the cardinal vein during lymphangiogenesis [44]. However, the poor suitability of mouse embryos for live imaging at the stages examined precludes unequivocal proof that LECs exit from the dermal capillary wall to form clusters via lymphvasculogenesis in addition to undergoing lymphangiogenesis. As intersomitic veins also exist at the timepoints examined in the study, LECs in the cervical and thoracic dermis could also, at least in part, arise by sprouting from venous endothelium to complement the dermal capillary-derived LEC pool. Notably, another study showed that only 70% of LECs in the lumbar dermis were labelled by *Tie2-Cre* [79]. It is not known whether the discrepancy between both studies is explained by variable activity of the *Tie2-Cre* transgene or the reporter alleles [137]. Alternatively, there may be rostrocaudal heterogeneity, whereby some LECs in the lumbar dermis region selectively derive from a lineage that does not express *Tie2*. Accordingly, further work is required to reconcile these differences.

## Future directions

Lymphvasculogenesis was recently identified as an alternative and complementary cellular mechanism to lymphangiogenesis during the formation of the lymphatic vascular system. Moreover, a wide range of diverse cell lineages have been suggested to contribute LECs to the lymphatic vasculature in different organs. Accordingly, it is now pertinent to investigate the relative importance and interplay between lymphvasculogenesis and lymphangiogenesis, both for lymphatic development, and also to define how diverse lymphatic origins may impact on morphological and functional lymphatic heterogeneity in different organs for health and disease. For some cell types, lineage origins have been shown to impact on cellular function. For example, macrophages emerge in sequential waves from multiple distinct lineages, whereby lineage origin dictates whether macrophages become tissue-resident or differentiate from circulating monocytes [138]. However, it is not yet known whether differences in the cellular origin of LECs contribute to lymphatic vessel heterogeneity. Alternatively, a wide variety of lineages may be employed to generate LECs, simply to increase their overall number or to improve organ colonisation. Another key question is to what extent findings from model organisms can be extrapolated to humans, given that only a few studies have investigated human lymphatic development to date [43, 139–141], likely owing to the limited availability of human fetal tissues. Below, we propose how several emerging technologies might provide a means to help close current gaps in our knowledge of lymphatic development.

### Ex vivo approaches

To date, our understanding of the cellular dynamics in mammalian lymphatic development is largely based on interpretation of static images from immunolabeled mouse or human tissues combined with extrapolation from zebrafish. Explant culture of developing organs may provide a novel approach to directly investigate such dynamics in mammals. For example, it has been demonstrated that isolated clusters of LECs are present in explanted E14.5 mouse kidney [43] and heart [103] and survive in tissue culture for several days. Accordingly, their coalescence into lymphatic vessels might be observed by live imaging ex vivo, especially when combined with advances in live imaging for deeper laser light penetration into tissues, such as multiphoton microscopy and light-sheet microscopy [142]. As precedence for the utility of this approach, it was shown that transplanting embryonic mouse cardiac mesothelium, but not epicardium, onto the ventral surface of explanted mouse embryo hearts gave rise to LEC clusters; a finding suggesting that some, but not all, cardiac tissues contain progenitors for lymphvasculogenesis [103]. The development of bioreactors or microfluidic systems [143] to better mimic blood flow and interstitial fluid accumulation [144] may further enhance lymphatic morphogenesis in such experiments. Ex vivo organ culture may also help to identify molecules required for lymphvasculogenesis, because the molecular regulation of this process remains poorly understood relative to lymphangiogenesis. For example, recombinant VEGFC increases the number of lymphatic clusters in explanted E14.5 mouse kidneys [43]. A tissue culture setting also provides relatively simple means for targeted genetic manipulation, for example, by photoactivation [145, 146] of explanted mammalian organs, analogous to the Kaede system used to study lymphatic development in zebrafish.

### Single cell technologies

Despite tremendous progress in defining the cellular lineages that give rise to lymphatics, identifying candidate progenitors and suitable promoters to drive Cre expression has traditionally relied on deduction or serendipity. Moreover, molecular heterogeneity between organ LECs remains poorly understood, especially when compared to the increasing knowledge of blood vascular heterogeneity. High throughput single cell approaches may provide an alternative, unbiased and complementary method for identifying and characterising LEC progenitors from different lineages, to compare LECs in different parts of the lymphatic tree and to investigate organ-specific LEC differentiation. In particular, single-cell RNA sequencing is a rapidly evolving technology that can be used to sequence the transcriptomes of individual cells from dissociated tissues, enabling the identification of different cell (sub)-types and their transcriptional states whilst also allowing the inference of cell transitions to predict lineage relationships. For example, an inferred differentiation ‘trajectory’, which can be obtained by plotting a gradient of gene expression [147] or the ratio of spliced to unspliced mRNA molecules [148] for transcriptionally related cell types along an axis, is used to describe ‘pseudotime’ [149]. This method, in turn, may help to infer LEC lineages, as has previously been done for other cell types. Alternatively, natural mutations, such as those occurring in mitochondrial DNA [150] or the introduction of heritable barcodes [151] could be used to retrospectively predict lymphatic lineage by computational approaches and is a technique which could also be applied to human tissue samples. The advent of spatial transcriptomics [152] may significantly enhance our insights into organ-specific lymphatic formation. For example, to determine the cellular and molecular composition of niches that promote the development of LECs in different organs. Transcription, however, does not necessarily indicate protein expression and, therefore, the development of single cell proteomic technologies will be important to understand functional heterogeneity of LECs in different organs [153].

### Intersectional genetics

Despite the identification of multiple lymphatic lineages, their functional relevance is challenging to establish. In particular, Cre expression in other, unwanted cell types complicates the interpretation of Cre-mediated lineage tracing and hinders the ability to ablate candidate genes specifically in LECs. For example, deletion of second heart field-derived lymphatics by knockout of *Prox1* using *Isl1-Cre* results in agenesis of cardiac ventral lymphatic vessels, but also defects in the dorsal lymphatics [99] that are not directly targeted by *Isl1-Cre* [99, 103]; this could be explained by a requirement for PROX1 in the myocardium that also arises from an *Isl1* lineage [154] and may regulate lymphatic development. To increase the specificity of targeting organ-specific populations of cells in the mouse, two different fragments of Cre might be expressed from promoters with overlapping expression, so that complementation causes the full Cre protein to be selectively expressed in cells that activate both promoters [155]. Alternatively, two different site-specific recombinases could be used in sequence or in parallel, for example, the Cre-loxP system together with the Dre-Rox system, which uses the Dre recombinase from the D6 bacteriophage and its target recombination sites, termed ‘Rox’ [156]. For example, a *Prox1-RSR-CreER*^*T2*^ mouse has recently been described, in which Dre expression is required to activate CreER^T2^ in *Prox1*^+^ cells [157]. As such, mating this line with an appropriate Dre recombinase line that is active in the second heart field could facilitate genetic recombination in the cardiac LEC lineage. This approach thereby surmounts the caveats of single promoter-based approaches. Moreover, intersectional genetics could be applied for targeted cell ablation approaches, for example by expressing diphtheria toxin [158] or its receptor [159] in LECs from different origins, to distinguish roles for venous- and non-venous-derived LECs in lymphatic vessels during development, or thereafter, in health and disease.

## Conclusion

The acquisition of knowledge of the cellular dynamics and origins of LECs in embryonic development is rapidly progressing. With the help of classical and emerging technologies, we anticipate a better understanding of lymphangiogenesis and lymphvasculogenesis and the origin of lymphatic lineages. Ultimately, an improved understanding of lymphatic development should significantly advance the study of lymphatic function in health as well as in disease, and potentially inform therapeutic approaches and regenerative medicine to treat the wide spectrum of diseases in which lymphatic vessels have been implicated.
